# Personality traits influence the effectiveness of hypomania checklist-32 in screening for bipolar disorder

**DOI:** 10.3389/fpsyt.2022.919305

**Published:** 2022-07-15

**Authors:** Qiang Wang, Aiping Sui, Lin Gong, Mohammad Ridwan Chattun, Ruichen Han, Qiuyun Cao, Diwen Shen, Yuzhe Zhang, Peng Zhao

**Affiliations:** ^1^Department of Medical Psychology, Nanjing Drum Tower Hospital, The Affiliated Hospital of Nanjing University Medical School, Nanjing, China; ^2^Jinling Institute of Technology, Nanjing, China; ^3^Department of Psychology, The Air Force Hospital from Eastern Theater of People’s Liberation Army (PLA), Nanjing, China; ^4^Department of Psychiatry, The Affiliated Brain Hospital of Nanjing Medical University, Nanjing, China; ^5^Nanjing Drum Tower Hospital, Clinical College of Jiangsu University, Nanjing, China

**Keywords:** bipolar disorder, major depressive disorder, personality traits, Eysenck Personality Questionnaire, hypomania checklist-32

## Abstract

**Background:**

It is clinically challenging to distinguish bipolar disorder (BD) from major depressive disorder (MDD) in the early stages. While the hypomania checklist-32 (HCL-32) is a proper auxiliary tool that is useful to differentiate between BD and MDD, there is currently no standard cut-off value. The variations in HCL-32 cut-off values could potentially be influenced by personality traits. Therefore, the aim of this study is to explore the effect of personality traits on the screening performance of HCL-32.

**Methods:**

In this retrospective cross-sectional study, 168 patients with BD or MDD were evaluated with the Eysenck Personality Questionnaire (EPQ) and HCL-32. The associations between demographic data, diagnosis and clinical rating scales were analyzed.

**Results:**

Diagnosis was not associated with extraversion but was related to neuroticism. HCL-32 scores in typical extraverted patients were higher in contrast to atypical extraverted patients. The best cut-off value for BD recognition of typical and atypical extraversion groups were 15 and 12.5, respectively. In patients with MDD, HCL-32 score of typical neuroticism was higher than the atypical type, but there was no difference in patients with BD. In typical neuroticism, there was no difference in HCL-32 scores between patients with MDD and BD. But among atypical neurotic patients, HCL-32 scores of BD were higher compared to MDD, with a cut-off value of 14.5.

**Limitations:**

This study had a small sample size.

**Conclusion:**

HCL-32 scores were affected by personality traits, with higher scores for typical extraversion and neuroticism. Clinicians should also consider the patients’ personality traits when referring to HCL-32 scores, so as to increase the recognition rate of BD and eliminate false positives.

## Introduction

While major depressive disorder (MDD) and bipolar disorder (BD) are both mood disorders, they are clinically distinct psychiatric illnesses (1). Since BD patients usually suffer from depressive episodes in the initial course of the disease, it is clinically difficult to distinguish BD from MDD (2). A nationwide study in China reported that approximately 20% of patients with BD are initially misdiagnosed with MDD (3). A misdiagnosis will delay the appropriate treatment and hence prolong the suffering of the patient and worsen the prognosis (4).

The hypomania checklist-32 (HCL-32) is a scale that screens for hypomanic symptoms and is useful to discriminate between BD and MDD (5), but its optimal cut-off value, sensitivity and specificity are still not completely homogenous. A study based in primary health care showed that 15 is the best cut-off value for distinguishing BD and MDD (6). Other studies have demonstrated that 14 is the ideal cut-off value, but there are variations in its sensitivity and specificity, for instance, in Europe (sensitivity 0.8, specificity 0.51) (7), Taiwan (sensitivity 0.82, specificity 0.67) (8) and China Mainland (sensitivity 0.69, specificity 0.98) (9). Another study validated that an optimal cut-off value of 13 could distinguish patients with BD-II from MDD, with a sensitivity of 0.77 and a specificity of 0.62 (1). Not only has the cut-off value of HCL-32 been found to be inconsistent in previous studies but, in clinical practice, some BD patients have low HCL-32 scores while MDD patients have high scores. The inconsistencies in the cut-off values of HCL-32 could potentially be explained by different personality traits.

Personality refers to the characteristic sets of behaviors, cognitions, and emotional patterns that are acquired through learning and habits (10). It is widely accepted that “The Big Five Personality Traits” comprise of extraversion, neuroticism, openness, agreeableness and conscientiousness (11). However, according to Hans Eysenck, the three major dimensions of personality that account for most of the variance in personality are extraversion, neuroticism, psychoticism (12). Both the “three-factor model” and “the five-factor model” are widely accepted approaches which extensively make use of self-report questionnaires to investigate personality (13). In personality theory, neuroticism is characterized by the disposition to experience negative emotional states (14). Extraversion is described as being talkative, outgoing and having a positive affect with very high levels of arousal (15). Psychoticism is not only associated with the liability to have a psychotic episode but also with aggressivity, impulsivity and sensation-seeking (16). To date, the most widely studied core personality traits that associated with BD and MDD are neuroticism and extraversion (17).

A study that investigated whether personality traits could predict the onset of depressive or manic episode found that both of the two episodes were associated with neuroticism and extraversion (18). Another study which explored the levels of neuroticism or extraversion between BD and MDD patients revealed that a high neuroticism might indicate a vulnerability to both BD and MDD patients (19). Personality traits in BD were characterized by high neuroticism as well as low extraversion (20). Compared to patients with BD I, BD II patients had higher neuroticism and lower extraversion (21). For depression, it was previously reported that extroversion was a protective factor while neuroticism was a risk factor (22). These findings suggest that the two traits of neuroticism and extraversion in personality traits have a strong influence on the course and outcome of both MDD and BD.

The aim of this study is to explore the effect of the two traits of neuroticism and extraversion in personality on the HCL-32 score in patients with MDD and BD. We hypothesized that personality traits might interfere with the screening performance of HCL-32. The importance of the current study is to improve the early clinical recognition of BD and reduce the misdiagnosis rate with MDD.

## Materials and methods

### Participants

In this retrospective cross-sectional study, 168 patients were recruited from Nanjing Drum Tower Hospital, The Affiliated Hospital of Nanjing University Medical School from December 2020 to October 2021. The participants were evaluated and diagnosed by one consultant psychiatrists according to the Diagnostic and Statistical Manual of Mental Disorders, fifth Edition (DSM-V) criteria. This study was approved by the Research Ethics Board of the Nanjing Drum Tower Hospital, the Affiliated Hospital of Nanjing University Medical School. This study is retrospective, and the risk to the subjects is not greater than the minimum risk, so informed consent was abandoned.

All patients met the following inclusion criteria: (1) MDD or BD; (2) 16 years old and above; (2) Han Chinese; (3) the ability to understand the meaning of each section of the rating scale. Patients who were diagnosed with other psychotic disorders including schizophrenia were excepted. Subjects who presented with comorbid psychiatric illnesses, alcohol or substance use disorders, were pregnant or had severe somatic diseases were also excluded.

### Psychological rating scales

The clinical data and two psychological scales data were retrospectively collected from electronic medical records. The demographic information included age, gender, education, clinical diagnosis.

In the current study, the Eysenck Personality Questionnaire (EPQ) was administered to both MDD and BD patients in order to examine personality traits (23). Only the two personality traits of extraversion and neuroticism were calculated. Extraversion scores higher than 61.5 was defined as typical extraversion, otherwise it was atypical extraversion. Among the atypical extraversion group, scores higher than 38.5 was defined as extraversion-intermediate, otherwise it was typical introversion. Similarly, if the neuroticism score was higher than 61.5, it was defined as typical neuroticism (24). Else, the scores were recognized as atypical neuroticism.

All participants were assessed with the self-administered HCL-32 questionnaire. Afterward, patients were divided into two groups according to their HCL-32 score. A score ≥14 was considered as a HCL positive group and a score <14 was regarded as a HCL negative group.

### Statistical analysis

Continuous data are presented as mean ± standard deviation and categorical data are presented as percentage (%). Independent two-sample *t*-test was used for age and each personality trait score of EPQ while rank sum test was utilized for education, and Chi-square test or Fisher’s exact test was employed for gender and diagnosis. Two-factor analysis of variance was used to explore the impact of diagnosis and personality traits on HCL-32 scores. ROC curve analysis was computed to find the best HCL-32 cut-off value under different personality traits. The HCL-32 score corresponding to the maximum value of “sensitivity + specificity −1” was used as the cutoff value. All statistical analyses were conducted using SPSS 21.0 software. All statistical tests were two-tailed, and *p* < 0.05 was considered statistically significant. As for multiple comparisons, Bonferroni correction were applied, the *p*-value output by the SPSS software was the calculated probability *p* multiplied by the number of comparisons, so that as long as the *p*-value was less than 0.05, the correction was passed.

## Results

### Description of the study sample

There were 168 patients included in the study. All subjects were divided into HCL positive (*N* = 104) and HCL negative (*N* = 64) groups. The demographic and clinical characteristics of the subjects are summarized in Tables 1, 2. In addition, see Table 3 for HCL-32 scores of subjects with different personality traits and diagnoses.

**TABLE 1:** Demographic characteristics and psychological scale results of patients.

Clinical characteristics	HCL positive (N = 104)	HCL negative (N = 64)	*P*-value
Age	23.8 ± 5.6	25.0 ± 6.9	0.26*^a^*
Female	71 (68.3%)	44 (68.6%)	0.95*^b^*
Education	87.3	79.9	0.29*^c^*
HCL-32	21.0 ± 4.2	7.81 ± 3.1	
Extraversion	57.7 ± 6.7	54.0 ± 6.7	0.001*^a^*
Neuroticism	58.0 ± 6.4	53.8 ± 7.2	<0.001*^a^*
Diagnosis			<0.001*^b^*
MDD	37 (35.6%)	49 (76.6%)	
BD	67 (64.4%)	15 (23.4%)	

HCL-32, the 32-item hypomania checklist; EPQ, the Eysenck Personality Questionnaire; MDD, major depressive disorder; BD, bipolar disorder.

^a^Two-sample t-test. ^b^Chi-square test. ^c^Rank sum test.

**TABLE 2:** Number of subjects with different personality traits.

Personality traits	Diagnosis		
	
	All patients (168)	MDD (86)	BD (82)
Extraversion			
Typical introversion	6	5	1
Extraversion-intermediate	124	73	51
Typical extraversion	38	8	30
Neuroticism			
Atypical neuroticism	132	74	58
Typical neuroticism	36	12	24

**TABLE 3:** Hypomania checklist score of each group.

Personality traits	Diagnosis		
	
	All patients	MDD	BD
Extraversion			
Typical introversion	14.0 ± 6.9	14.8 ± 7.4	10 ± 0
Extraversion-intermediate	14.3 ± 6.9	11.9 ± 6.6	17.7 ± 5.8
Typical extraversion	21.8 ± 6.4	15.3 ± 8.3	23.6 ± 4.6
Neuroticism			
Atypical neuroticism	14.9 ± 7.3	11.5 ± 6.3	19.3 ± 5.9
Typical neuroticism	19.7 ± 6.9	17.8 ± 7.3	20.6 ± 6.7

### Comparisons between hypomania checklist positive and hypomania checklist negative groups

There were statistically significant between-group differences in diagnosis (χ^2^ = 26.636, *p* < 0.001), extraversion (*t* = 3.456, *p* = 0.001), and neuroticism (*t* = 4.001, *p* < 0.001). The differences in age (*t* = 1.128, *p* = 0.261), education (*z* = 1.067, *p* = 0.29), and gender (χ^2^ = 0.004, *p* = 0.948) between the two groups were not statistically significant. See Table 1.

### Two-factor analysis of variance

#### Extraversion and diagnosis

**FIGURE 1:**
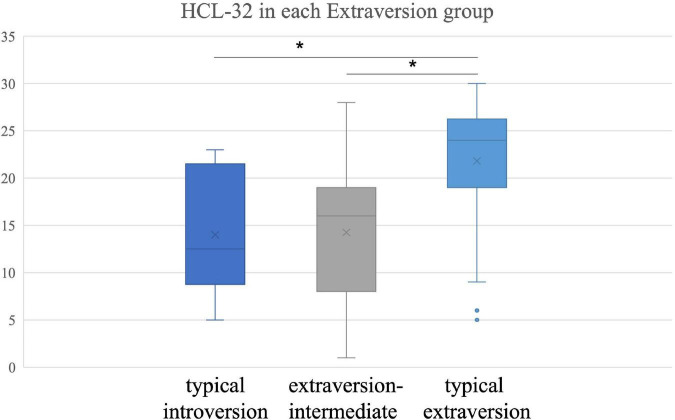
Hypomania checklist-32 score in each Extraversion group. *p < 0.05 Bonferroni correction.

There was no interaction between extraversion and diagnosis on the HCL-32 score (*F* = 1.764, *p* = 0.175, partial η^2^ = 0.021). An increase in extraversion score led to higher overall HCL-32 scores. Patients exhibiting typical extraversion scored 7.56 ± 1.1 points higher than the extraversion-intermediate group (*p* < 0.001, Bonferroni correction) and 7.82 ± 2.7 points higher than the typical introversion group (*p* = 0.013, Bonferroni correction) (Figure 1), but there were no differences between the extraversion-intermediate and typical introversion groups.

#### Neuroticism and diagnosis

**FIGURE 2:**
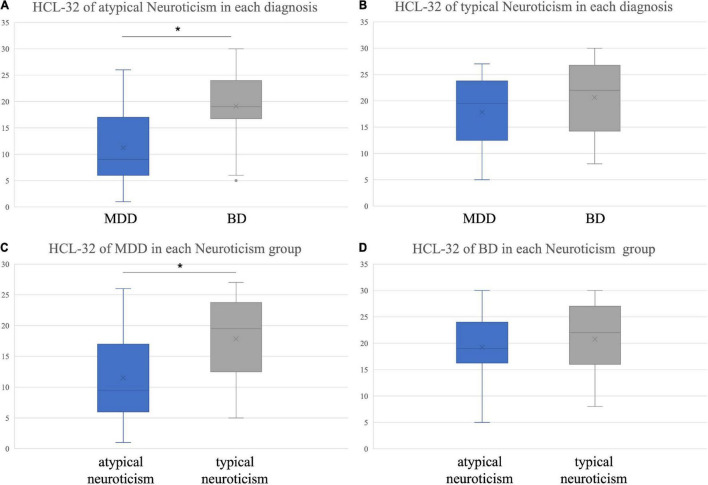
Hypomania checklist-32 score of different personality traits in each diagnosis. **(A)** Differences in HCL-32 scores between BD and MDD patients in the atypical neuroticism group; **(B)** Differences in HCL-32 scores between BD and MDD patients in the typical neuroticism group; **(C)** Differences in HCL-32 scores between atypical and typical neuroticism in MDD patients; **(D)** Differences in HCL-32 scores between atypical and typical neuroticism in BD patients. *p < 0.05 Bonferroni correction.

There was an interaction between neuroticism and diagnosis on the impact of HCL-32 score (*F* = 4.144, *p* = 0.043, partial η^2^ = 0.025). When neuroticism was atypical, HCL-32 scores of distinct diagnosis were different (*F* = 50.484, *p* < 0.001); BD score was 7.86 ± 1.1 higher than MDD score (*p* < 0.001, Bonferroni correction) (Figure 2A). Conversely, when neuroticism was typical, there was no statistically significant difference in the HCL-32 scores of the two diagnoses (Figure 2B).

In patients with MDD, the difference in HCL-32 scores between the two types of neuroticism was statistically significant (*F* = 10.458, *p* = 0.001), and the typical neuroticism score was 6.35 ± 2.0 higher than the atypical type score (*p* = 0.001, Bonferroni correction) (Figure 2C). However, in patients with BD, the difference in HCL-32 scores between the two types was not statistically significant (*F* = 0.699, *p* = 0.404) (Figure 2D).

### ROC analysis

**FIGURE 3:**
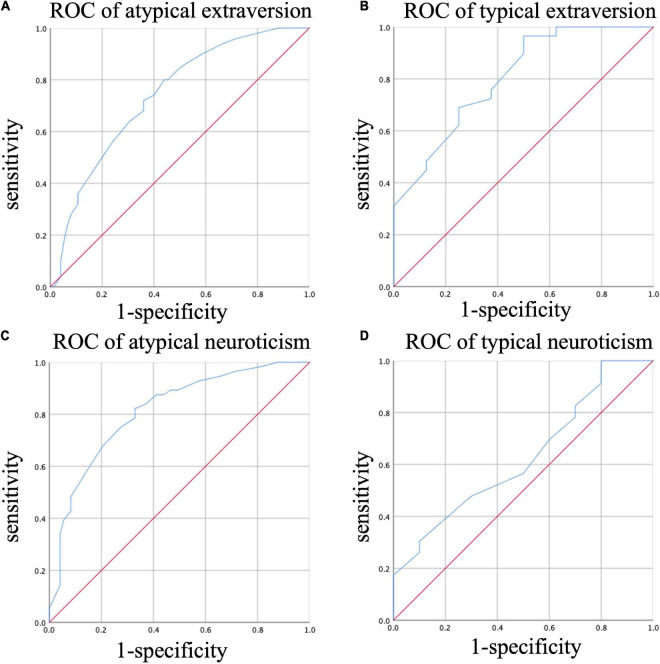
ROC of HCL-32 for different diagnosis in different personality traits. **(A)** ROC analysis of the ability of HCL-32 to discriminate between MDD and BD in atypical extraversion group; **(B)** ROC analysis of the ability of HCL-32 to discriminate between MDD and BD in typical extraversion group; **(C)** ROC analysis of the ability of HCL-32 to discriminate between MDD and BD in atypical neuroticism group; **(D)** ROC analysis of the ability of HCL-32 to discriminate between MDD and BD in typical neuroticism group.

According to extraversion, patients were divided into typical extraversion and atypical extraversion groups. The best cut-off value of HCL-32 in the atypical extraversion group for identifying BD was 12.5 (AUC = 0.74, *p* < 0.001, sensitivity = 0.8, specificity = 0.56) (Figure 3A), and the best cut-off value of HCL-32 in the typical extraversion group was 15 (AUC = 0.80, *p* = 0.009, sensitivity = 0.97, specificity = 0.5) (Figure 3B).

When neuroticism was atypical, the best cut-off value of HCL-32 for identifying BD was 14.5 (AUC = 0.81, *p* < 0.001, sensitivity = 0.82, specificity = 0.67) (Figure 3C). However, when neuroticism was typical, ROC curve analysis showed no positive results (AUC = 0.63, *p* = 0.248) (Figure 3D). The above results were also shown in Table 4.

**TABLE 4:** ROC index of each group.

Group	AUC	*P*-value	Sensitivity (%)	Specificity (%)	Cut-off value
Atypical extraversion	0.80	0.009	0.97	0.5	15
Typical extraversion	0.74	<0.001	0.8	0.56	12.5
Atypical neuroticism	0.81	<0.001	0.82	0.67	14.5
Typical neuroticism	0.63	0.248	−⁣−	−⁣−	–

## Discussion

In the present study, HCL-32 scores were related to different personality traits reflected in the EPQ scale. While there was no association between extraversion and diagnosis, extraversion had an impact on HCL-32 scores. A higher extraversion score contributed to higher overall HCL-32 scores. In addition, the HCL-32 score of typical extroverted patients was significantly higher compared to intermediate and typical introverted patients. Moreover, diagnosis was associated with neuroticism. When neuroticism was typical, there was no difference in HCL-32 scores between patients with MDD and BD. Although there was no difference in the HCL-32 scores of BD patients with different neurotic types, the scores of typical neurotic patients were higher than those of atypical neurotic patients in MDD patients.

Previous studies have shown that HCL-32 is related to personality which is consistent with the results of the current study. However, these studies only provided qualitative results. A study which investigated the temperament and bipolar features in depressed patients found that elevated neuroticism resulted in an increase in Beck Depression Index (BDI) score which was positively correlated with HCL-32 score (25). This finding suggested that neuroticism is associated with a high HCL-32 score. A previous study which assessed the association between symptoms of mood disorders and personality traits *via* the Big Five Personality Questionnaire revealed that extraversion was the most reliable predictor of hypomania symptoms assessed by HCL-16 and neuroticism was positively correlated with the hypomanic symptoms of the Mood Disorder Questionnaire (MDQ) scale (26). Another study that examined personality traits in patients with postpartum hypomania reported that extraversion on the EPQ scale was significantly associated with an increased risk of hypomanic symptoms as assessed by HCL-32 scale (27). The abovementioned findings are in line with the results of the current study which illustrated that HCL-32 scores are affected by distinct personality traits.

In our study, diagnosis was not associated with extraversion but was linked to neuroticism. Previous studies on personality traits differences in BD, MDD and the general population revealed that patients with MDD and BD have higher levels of neuroticism but lower extraversion compared with the general population (19, 22). Since patients with BD and MDD usually experience negative emotional states, it is not surprising that these patients have neuroticism traits. On the other hand, the HCL-32 score of typical extraverted patients was higher than those of typical introverted and extraversion-intermediate patients. When ROC analysis was performed, increasing the HCL-32 score of typical extraverted patients to 15 points was more beneficial in eliminating false positives while reducing the HCL-32 score of atypical extraverted patients to 12.5 points was beneficial in enhancing BD recognition rate. When neuroticism was typical, there was no difference in HCL-32 scores between MDD and BD, suggesting that the HCL-32 discrimination ability was not suitable for patients with this type of personality. When neuroticism was atypical, the HCL-32 score of MDD patients was lower than that of BD patients, and the recommended cut-off value was 14.5 points.

To the best of our knowledge, this is the first research to explore the interaction of personality traits *via* EPQ scale and HCL-32 scores, and quantify the results to provide clinicians with specific reference values to distinguish between BD and MDD. The results showed that HCL-32 needs to be combined with the assessment of patients’ personality traits.

## Conclusion and limitations

In summary, the results of this study could provide clinicians with more practical and numerical reference indicators of HCL-32 scale, which are conducive to early clinical identification of BD patients. In the future, clinicians should also consider the patients’ personality traits when referring to HCL-32 scores, so as to increase the recognition rate and eliminate false positives as much as possible. The main limitation of this study is that sample size is small. A larger and more representative sample is needed for validation of our results. Moreover, a post-morbid change of personality in patients with BD and MDD has not been thoroughly assessed and cannot be completely excluded. Since all the scales used in the current study are self-rating scales, there is the possibility of a bias.

## Data Availability Statement

The original contributions presented in this study are included in the article/supplementary material, further inquiries can be directed to the corresponding author.

## Ethics statement

The studies involving human participants were reviewed and approved by the Research Ethics Board of the Nanjing Drum Tower Hospital, the Affiliated Hospital of Nanjing University Medical School. Written informed consent from the participants’ legal guardian/next of kin was not required to participate in this study in accordance with the national legislation and the institutional requirements.

## Author contributions

QW and AS: conceptualization, data curation, formal analysis, and writing original draft. LG, RH, DS, and YZ: data curation. MC: writing original draft. PZ and QC: conceptualization. All authors contributed to the article and approved the submitted version.

## Conflict of Interest

The authors declare that the research was conducted in the absence of any commercial or financial relationships that could be construed as a potential conflict of interest.

## Publisher’s Note

All claims expressed in this article are solely those of the authors and do not necessarily represent those of their affiliated organizations, or those of the publisher, the editors and the reviewers. Any product that may be evaluated in this article, or claim that may be made by its manufacturer, is not guaranteed or endorsed by the publisher.
